# Supplementary material for: Effects of Psychological Interventions on Performance Anxiety in Performing Artists and Athletes: A Systematic Review with Meta-Analysis
Source: Behav Sci (Basel). 2023 Nov 7;13(11):910. doi: 10.3390/bs13110910 (PMC10669558; doi:10.3390/bs13110910)
Supplement: Supplementary file 1 [file behavsci-13-00910-s001.zip › Table S2.pdf]

behavioral sciences

MDPI

Table S2. Scottish Intercollegiate Guidelines Network Methodology checklist for randomised controlled trials.

	Braden et al. (2015)	Clark & Williamon (2011)	Georgakaki & Karakasidou (2017)	Hatzigeorgiadis et al. (2009)	Hoffman & Hanrahan (2012)	Marshall & Gibson (2017)	Osborne et al. (2007)	Terry et al. (1995)	Thurber et al. (2010)	Whitaker (1984)	Wolch et al. (2021)	Yahya et al. (2016)	Veskovic et al. (2019)	Kerr et al. (1993)	Dehghani et al. (2018)	Fortes et al. (2016)	Mehrsafar et al. (2019)	Kanniyan. (2014)	Grobbelaar (2018)	Spahn et al. (2016)
1.1 The study addresses an appropriate and clearly focused question.	Yes	Yes	Yes	Yes	Yes	Yes	Yes	Yes	No	Yes	Yes	Yes	Yes	Yes	Yes	Yes	Yes	Yes	Yes	Yes
1.2 The assignment of subjects to treatment groups is randomised.	Yes	No	Yes	Yes	Yes	Yes	Yes	Yes	Yes	No	Yes	Yes	Yes	Yes	Yes	Yes	Yes	Cs	Yes	No
1.3 An adequate concealment method is used.	No	No	No	No	No	No	No	No	No	No	No	No	No	No	No	No	No	No	No	No
1.4 The design keeps subjects and investigators 'blind' about treatment allocation.	Cs	No	No	No	No	No	No	Cs	Cs	No	No	Cs	No	No	No	No	No	No	Cs	No
1.5 The treatment and control groups are similar at the start of the trial.	Yes	No	Yes	Yes	No	No	No	Yes	Yes	Yes	Yes	Cs	Yes	No	Yes	Yes	Yes	Yes	Yes	No
1.6 The only difference between groups is the treatment under investigation.	Yes	Yes	Cs	Yes	Yes	Yes	No	Yes	Cs	Yes	Yes	Cs	Yes	Yes	Cs	Yes	Yes	Yes	Yes	No
1.7 All relevant outcomes are measured in a standard, valid and reliable way.	Yes	Yes	Yes	Yes	Yes	Yes	Yes	Yes	Yes	Yes	Yes	Yes	Yes	Yes	Yes	Yes	Yes	Yes	Yes	Yes
1.8 What percentage of the individuals or clusters recruited into each treatment arm of the study dropped out before the study was completed?	0%	19%	0%	0%	3%	0%	0%	0%	30%	0%	0%	0%	0%	0%	0%	8%	0%	0%	13%	13%
1.9 All the subjects are analysed in the groups to which they were randomly allocated (often referred to as intention to treat analysis).	Yes	Yes	Yes	Yes	Yes	Yes	Yes	Yes	Yes	Yes	Yes	Yes	Yes	Yes	Yes	Yes	Yes	Yes	Yes	Yes
1.10 Where the study is carried out at more than one site, results are comparable for all sites.	Na	Na	Na	Na	Na	Na	Na	Na	Na	Na	Na	Na	Na	Na	Na	Na	Na	Na	Na	Na
2.1 How well was the study done to minimise bias? Code as follows: High quality (++), Acceptable quality (+), Low quality (-)	++	+	+	++	+	+	-	+	-	-	++	+	++	+	+	++	++	+	+	-
2.2 Taking into account clinical considerations, your evaluation of the methodology used, and the statistical power of the study, are you certain that the overall effect is due to the study intervention?	Yes	Yes	Yes	Yes	Yes	Yes	Yes	Yes	Yes	Yes	Yes	Yes	Yes	Yes	Yes	Yes	Yes	Yes	Yes	Yes

2.3 Are the results of this study directly applicable to the patient group targeted by this guideline?	Yes	Yes	Yes	Yes	Yes	Yes	Yes	Yes	Yes	Yes	Yes	Yes	Yes	Yes	Yes	Yes	Yes	Yes	Yes	Yes
Response Options: Yes / No / Can`t say (Cs) / Not applicable (Na)																				
